# A Comparative Study on Changes in Protein, Lipid and Meat-Quality Attributes of Camel Meat, Beef and Sheep Meat (Mutton) during Refrigerated Storage

**DOI:** 10.3390/ani13050904

**Published:** 2023-03-02

**Authors:** Kusaimah Manheem, Oladipupo Adiamo, Ume Roobab, Khaja Mohteshamuddin, Hassan. M. Hassan, Nilesh. P. Nirmal, Sajid Maqsood

**Affiliations:** 1 Department of Food Science, College of Agriculture and Veterinary Medicine, United Arab Emirates University, Al Ain P.O. Box 15551, United Arab Emirates; 2 Centre for Nutrition and Food Sciences, Queensland Alliance for Agriculture and Food Innovation (QAAFI), The University of Queensland, Brisbane, QLD 4068, Australia; 3 Department of Veterinary Medicine, College of Agriculture and Veterinary Medicine, United Arab Emirates University, Al Ain P.O. Box 15551, United Arab Emirates; 4 Institute of Nutrition, Mahidol University, 999 Phutthamonthon 4 Road, Salaya, Nakhon Pathom 73170, Thailand

**Keywords:** camel meat, beef, mutton, refrigerated storage, protein characterisation, lipid oxidation

## Abstract

**Simple Summary:**

This study investigated a comparative analysis on changes in protein and lipid fractions and quality attributes of camel meat, cattle meat (beef) and sheep meat (mutton). The samples were studied over 9 days of refrigerated storage. Camel meat underwent higher lipid oxidation compared with beef and sheep meat. All meat types showed a decrease in the haem pigments and redness (a* values) indicating the oxidation of the haem pigment. Drip loss was high in camel meat and mutton compared with the beef samples. Camel meat was tougher, with higher hardness values, followed by beef and mutton samples, which decreased over 3 and 9 days of storage, indicating the degradation of structural proteins. In conclusion, the study provided valuable information on the properties of different red meats during storage and variations in their protein and lipid fractions and other quality attributes during storage.

**Abstract:**

An in-depth characterisation of protein and lipid fractions and changes in the physicochemical and meat-quality attributes of camel meat, beef and mutton over 9 days of refrigerated storage was investigated. The lipids of all the meat samples, especially those in camel meat, underwent significant oxidation in the first 3 days of storage. A decrease in pigment and redness (a* value) with an increase in the storage time was noticed in all the meat samples, suggesting the oxidation of the haem protein. The mutton samples displayed greater protein extractability, while the protein solubility values in all the meat samples were similar, and these varied as storage progressed. The drip loss percentage in camel meat and mutton were two times higher than in beef, and it increased during storage period. The textural properties of fresh camel meat were higher than mutton and beef, and these decreased during day 3 and 9, respectively, indicating the proteolysis and the degradation of the structural proteins, which were also evident from the SDS-PAGE pattern.

## 1. Introduction

Red meat is an important source of high-quality protein that provides all the essential amino acids [1] as well as several essential minerals, such as iron, zinc, copper and magnesium, that are required for optimal health and wellbeing [2]. Modern nutrition guidelines recommend reducing cholesterol and saturated fatty acid intake, which has subsequently affected consumers’ meat choice and preparation [3]. Camel meat is an inherently lean meat and has low fat content compared with other commercial red meats, such as beef and mutton [4]. Strong cultural and folklore beliefs that camel meat has medicinal benefits from its lower cholesterol concentration and relatively higher polyunsaturated fatty acids than beef have led to a higher preference for camel meat than meat from other domesticated species in several African and Middle Eastern countries [5,6,7].

Camels possess a unique ability to thrive and tolerate harsh environmental conditions (e.g., high ambient temperature, low/irregular feed and water) [8] and the ability to forage on plants that are not accepted by other livestock. Camels are an excellent choice for livestock production in arid and semiarid climatic conditions that are not suitable for other domesticated livestock [9]. With global warming and increased arid/semiarid conditions in many parts around the world, camel raising represents a realistic promise to alleviate the protein shortage in these regions. Camel meat is generally considered coarse and tough because of the slaughter-age effect, where background toughness and coarser muscle fibres become prevalent [10]. However, recent research has provided guidelines for opportunities to improve camel meat tenderness by using plant proteases [11].

Despite its potential as an alternative meat source in arid and semiarid regions, where camels can be produced more economically than cattle can, camel meat is not widely consumed and is often misunderstood to be of lower nutritional value and quality compared with other types of red meat. This highlights the need for further research to better understand the composition and stability of camel meat during storage to improve its acceptability and potential as a source of nutritious meat.

The majority of studies that have investigated camel meat have focused on its nutritional value (i.e., amino acid profile, fatty acid composition, cholesterol content and minerals content) compared with other meats to highlight its healthiness [4,8,12]. Few studies have investigated changes in the physicochemical properties of camel meat during refrigerated storage [4]. By reducing the rates of microbiological, chemical and biochemical changes, low-temperature storage helps to maintain the quality and freshness of meat. As a result, it is crucial to study and monitor the changes that occur in meat during low-temperature storage to ensure that its freshness and quality are maintained over time. There are no studies that have compared the quality and stability of camel meat with other red meats from the same farming background under similar storage conditions. Such comparative studies on changes in the protein and lipid characteristics of fresh camel meat compared with other common red meats such as beef (cattle meat) and mutton (sheep meat) during refrigerated storage are crucial to gaining a better understanding of camel meat stability during storage and any major issues that might affect its consumer acceptability. The objective of this study was to investigate changes in the protein and lipid compositions and the texture properties of camel meat compared with those in beef and mutton from the same farming background over 9 days of refrigerated storage at 4 °C.

## 2. Materials and Methods

### 2.1. Preparation of Meat Samples

Right and left semitendinosus (ST) muscles were excised from six carcasses, each from three animal species, namely camel (males, age: 3–4 years), cattle (males; age: 3–4 years) and sheep (males, age: 2–2.5 years), and they were used in a comparative study. The animals were slaughtered in a local slaughterhouse following the United Arab Emirates Standard No. 993/2000 for animal slaughtering, which covers the requirements for halal slaughtering. The camels (*Camelus dromedaries*) were of the Hizami breed, the sheep were of native origin (Najdi breed) and the cattle belonged to the Chianina breed. Animals were reared in the semi-intensive management system and fed ad libitum on a Rhodes grass (*Chloris gayana*) hay diet. The ST muscles were manually removed from the carcasses of animals at 24–36 h postmortem, during which the carcasses were stored at 4 °C. The samples were placed in polyethylene bags stored in insulated chilling boxes during transportation to laboratory, and the samples were processed within 2 h. Each muscle was sliced into 2 cm steaks that were randomly assigned to the various biochemical and texture measurements over a 9-day storage period at 4 °C. The samples were placed in polystyrene trays that were wrapped with commercially available cling film (Falcon Food Packaging, UAE). The samples were placed in a chiller at 4 °C, and the trays were randomly rotated during the storage time and sampled at the designated storage time.

### 2.2. Determination of Meat Quality during Storage

#### 2.2.1. The pH

About 10 g of the meat samples was homogenised with 50 mL of chilled distilled water, and the pH values were measured with a digital pH meter (OHAUS, Starter 3100, USA) following the PN-ISO 2917:2002 method.

#### 2.2.2. Drip Loss

A 10 g sample of meat was placed in a plastic bag, which was hung from a hook and stored at 4 °C for 48 h. The drip loss (%) was determined from the weight loss of the samples upon storage at 4 °C for 48 h, as described in [13]. After 48 h, the sample was removed from the bag, dried on absorbent paper and reweighed.

The percentage of change in weight was used to calculate the drip loss by using Equation (1):

(1) $$Drip\;loss\;\left(\%\right)=\frac{\left(weight\;of\;sample\;before\;storage-weight\;of\;sample\;after\;storage\right)}{weight\;of\;sample\;before\;storage}\times 100$$

#### 2.2.3. Colour

Colour of the ground meat samples was measured using a colorimeter (HunterLab, Model colour Flex, Reston, VIRG, USA) equipped with the light source illuminant D65 and 10° standard observer, and a port size of 0.50 inches was used. The colour was reported in the CIE colour L*a*b* system where L*-value is lightness, a*-value is redness/greenness and b* value is yellowness/blueness. Before each measurement, the apparatus was standardised against a white plate. The measurements were carried out on days 0, 3, 6 and 9 of storage at 4 °C.

#### 2.2.4. Textural Properties

Textural properties of samples were determined using a texture analyser (CT3-4500, Brookfield Engineering Laboratories, Middleboro, USA) with a cylindrical probe that had a diameter of 50 mm. Hardness, cohesiveness, springiness, gumminess and chewiness were calculated from the force–time curves generated for each sample on day 0 and day 4.

For determination of shear force values of different meat samples, cores (10 × 10 mm cross sections) were isolated from the centre of meat cubes and parallel to the muscle fibres using two scalpel blades at a fixed distance. Cores were sheared perpendicularly to the fibres in two places using a shear blade geometry attached to Brookfield texture analyser. Shear force measures the maximum force required to shear across the muscle fibre. Shear force was determined as the average of 6 peak force recordings across each muscle core.

### 2.3. Characterisation of Protein Fraction

#### 2.3.1. Protein Analysis by Sodium Dodecyl Sulphate Polyacrylamide Gel Electrophoresis (SDS-PAGE)

Ground meat samples (camel meat, beef and mutton) were subjected to SDS-PAGE according to the method in Laemmli [14], with a slight modification, as described by Maqsood and Benjakul [15]. The percentage of running gel and stacking gel was 10% and 4%, respectively, and 15 µg protein was loaded into each well.

#### 2.3.2. Determination of Protein Extractability

Protein extractability measurements determine the water-soluble and salt-soluble protein fractions. The measurements were carried out as described in a previous study [16]. Meat sample (2 g) was homogenised at a speed of 11,000 rpm for 1 min in 25 mL homogenisation buffer (contained 20 mM Tris-HCl containing 50 mM KCl, pH 7.0). The homogenised mixture was centrifuged at 5000× *g* for 20 min at 4 °C. The protein fraction separated in the resultant supernatant was defined as “water-soluble proteins”. The pellet was then homogenised in 30 mL of a high salt extraction buffer (contained 20 mM Tris-HCl containing 0.6 M KCl, pH 7.0). The homogenate was centrifuged at 5000× *g* for 20 min. The resultant supernatant was defined as “salt-soluble proteins”. The protein concentration in the supernatants was determined by using the Biuret method, using bovine serum albumin as a standard.

#### 2.3.3. Determination of Protein Solubility

Sarcoplasmic solubility and total protein solubility were determined according to the method of Hrynets et al. [17], as described by Maqsood et al. [4].

### 2.4. Determination of Lipid Oxidation Products

#### 2.4.1. Peroxide Value (PV)

Meat samples were subsampled at 0, 3, 6 and 9 days of storage at 4 °C, and the PV was analysed [18]. Knife-minced meat sample (1 g) was homogenised in 11 mL of chloroform/methanol (2:1, *v*/*v*) mixture using an Ultra-Turrax T25 homogeniser (IKA-Werke GmbH & Co., Staufen im Breisgau, Germany) at a speed of 13,500 rpm for 2 min. The PV was determined from a standard curve constructed using Cumene hydroperoxide at concentration range of 0.5–2 ppm. The PV was reported as mg of hydroperoxide/kg sample.

#### 2.4.2. Thiobarbituric Acid Reactive Substances (TBARS)

The TBARS values of meat samples were analysed using the method of Buege and Aust [19]. 1,1,3,3-tetramethoxypropane was used to generate a malondialdehyde (MDA) standard curve at a concentration range of 0 to 10 ppm. Each TBARS value was expressed as mg of MDA equivalents/kg sample.

### 2.5. Determination of Meat Pigments

#### 2.5.1. Total Haem Pigment Content Analysis

Total pigment contents in the camel meat, beef and mutton samples were determined using the method of Hornsey [20], with some modifications. About 10 g of the ground sample was mixed with 25 mL of acidified acetone solution (contained 40 mL of acetone, 9 mL of water and 1 mL of HCl), and the mixture was homogenised at 13,500 rpm for 15 s. Another 25 mL of the acidified acetone solution was added to the mixture, and the mixture was thoroughly mixed and allowed to stand in the dark for 1 h. Next, the mixture was centrifuged at 2200× *g* for 10 min at 4 °C using an Allegra X-30R centrifuge (Beckman Coulter, Inc, Brea, California, USA). The supernatant was filtered with Whatman No.1 filter paper, and the absorbance was measured at 640 nm against a reagent blank. The concentration of the total pigments in the samples was calculated by multiplying the absorbance by 6800 and dividing by the sample weight, and the total pigment content is reported as µg haematin/g meat [21].

#### 2.5.2. Haemoglobin Content Analysis

Haemoglobin content was determined according to following Richards and Hultin [18] and modifications suggested by Maqsood et al. [4]. The haemoglobin concentration was calculated using a millimolar extinction coefficient of 125 for oxyhaemoglobin at pH 8 [22] and expressed in mM.

#### 2.5.3. Myoglobin Content Analysis

Myoglobin content was determined as described by Maqsood et al. [4], using direct spectrophotometric measurement, and the myoglobin content was expressed as mg/g sample.

### 2.6. Statistical Analysis

All the experimental analyses were conducted using six meat portions (*n* = 6) for all animals. The experiment was designed by implementing a completely randomised design, where the fixed effects were the three types of meat. Moreover, meat samples within a muscle and muscle portion within a carcass were considered as the random effects. Data were statistically analysed by a one-way analysis of variance (ANOVA) using a general linear model (GLM) procedure considering meat type as the main effect and to compare the means of the different meat-quality parameters and analysis carried out among the three types of meats during storage. The effects of species and storage time on the measured parameters and the differences between means were evaluated by Duncan’s multiple range test, with a significance level of 0.05, using a SPSS package (SPSS 14.0 for Windows, SPSS Inc., Chicago, IL, USA).

## 3. Results

### 3.1. Changes in pH

**Figure 1:**
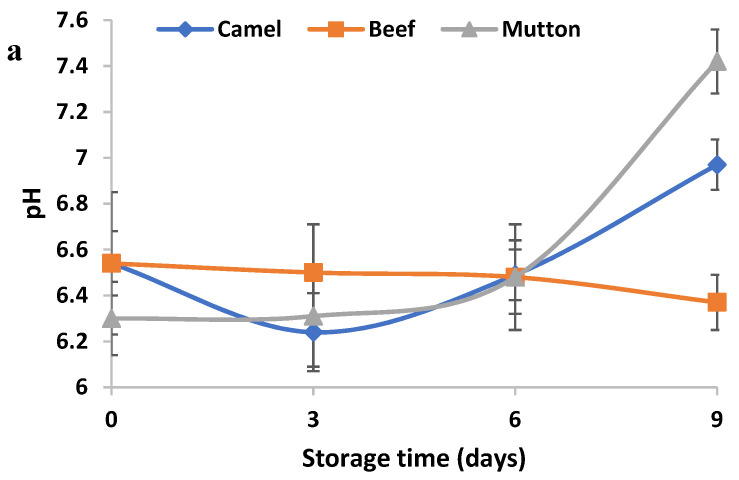

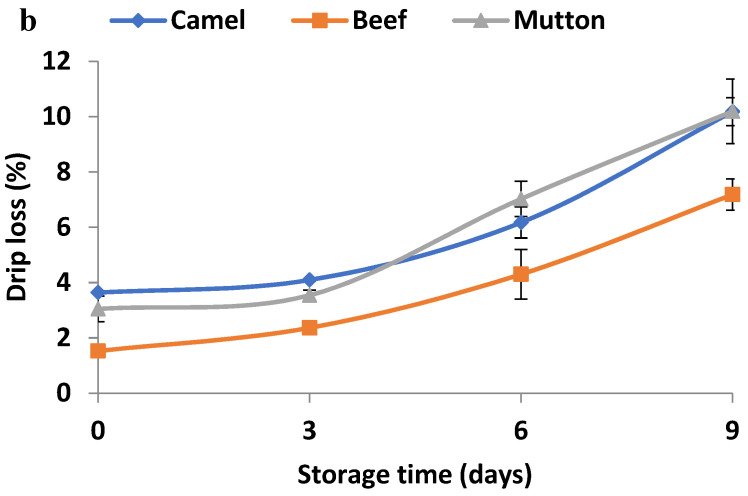
Changes in pH (**a**) and drip loss (%) (**b**) in camel meat, beef and mutton over 9 days of refrigerated storage. Data are represented as mean ± SEM (*n* = 6).

Mutton had a lower initial pH than that of beef and that of camel meat (Figure 1a). There were variations in the pH of the meat samples during storage. The pH of camel meat decreased after the first 3 days of storage and thereafter increased at day 9 of storage as compared with day 0. However, a nonsignificant reduction in pH was observed for the beef sample during storage. Meanwhile, the pH of the mutton sample (pH = 7.42) significantly increased at the end of storage and was reported to be highest among the meat samples (*p <* 0.05).

### 3.2. Changes in Drip Loss

The drip loss percentage in fresh camel meat and mutton were found to be significantly greater than that in beef (Figure 1b). A gradual rise in drip loss was observed in all the meat samples when the storage time increased (*p <* 0.05). At the end of the 9-day storage, the rates of drip loss in fresh camel meat and mutton were found to be higher than that in beef (*p* < 0.05).

### 3.3. Changes in Protein Extractability and Solubility

The fresh meat samples differed in the level of water-soluble protein (WSP) extractability and salt soluble protein (SSP) extractability (Table 1). A significantly higher WSP extractability was found in fresh beef and mutton as compared with camel meat (*p* <0.05). As storage progresses, increases in the WSP and SSP extraction in all the meat samples were observed. Camel meat showed significant increases in protein (WSP and SSP) extractability up to day 6. In contrast, increases in WSP extractability in beef samples were observed from day 3 to day 9, while SSP extractability increased throughout the days of storage. However, mutton showed a significant increase in both WSP and SSP at all the storage intervals as compared with day 0. At the end of the storage period, camel meat had significantly lower WSP and SSP values than beef or mutton. For the SSP extractability, initially from day 3 to day 6 of storage, mutton showed no significant difference, while towards the end of storage, significantly higher SSP extractability was noticed compared with camel meat and beef (*p* < 0.05). The protein solubility, both sarcoplasmic (SPS) and total protein (TPS), in camel meat and beef were similar but lower than that in mutton at day 0 (Table 1). As storage progressed, the SPS in the camel and beef meat samples significantly increased after 6 days of storage, whereas the mutton sample showed increases throughout all the days of storage. Similarly, the TPS in camel meat significantly increased on day 3; however, a significant increase in the TPS for the mutton and beef samples was observed on day 6.

### 3.4. Changes in Colour Parameters

Camel meat had lower lightness (L*) and higher redness (a*) values than mutton and beef (Table 1). However, fresh camel meat and fresh beef samples have higher yellowness (b*) values than mutton. As storage progressed, the lightness (L*) and yellowness (b*) of the meat samples decreased (*p <* 0.05) from day 6 of refrigerated storage—excluding the L* value of the beef samples, which decreased throughout the storage period (Table 1). However, all the meat samples displayed a significant (*p <* 0.05) and gradual decrease in a* values over the course of the 9 days of refrigerated storage. Moreover, the camel redness and yellowness values decreased more significantly than those of the other species (except for an increase in a* at day 9).

### 3.5. Changes in Protein Pattern by SDS-PAGE

**Figure 2:**
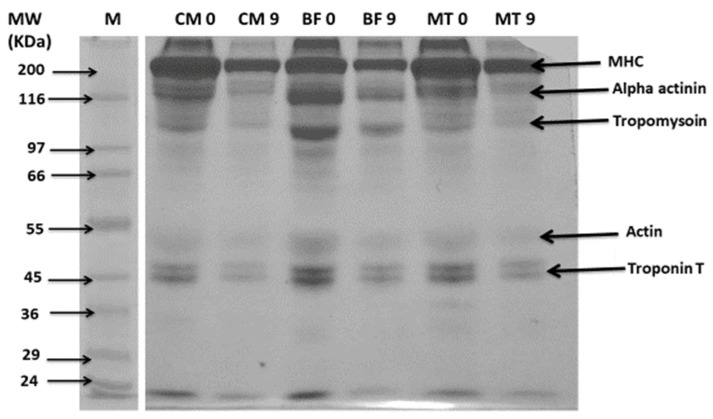
Sodium dodecyl sulphate polyacrylamide gel electrophoresis (SDS-PAGE) pattern of proteins extracted from camel meat, beef and mutton on day 0 and day 9 of refrigerated storage. Notes: CM 0 and CM 9; BF 0 and BF 9; and MT 0 and MT 9 denotes camel meat, beef and mutton on day 0 and on day 9 of storage, respectively.

The electrophoresis analyses of the protein changes in all the meat samples during the refrigerated storage of day 0 through day 9 has been shown in Figure 2. In general, during the start of the storage period, the protein pattern of all the meat samples showed clear visible bands of the major histocompatibility complex (MHC), alpha actinin, tropomyosin, actin and troponin T. At the end of storage, the band intensity significantly reduced for camel meat, and to a lesser extent for mutton meat and the smallest were for beef. The disappearance of bands corresponding to alpha actinin, tropomyosin and actin was noticed for the camel and mutton meat samples stored for 9 days. A significant degradation of the MHC was observed for all the meat samples after 9 days of storage.

### 3.6. Changes in Lipid Oxidation Products

**Figure 3:**
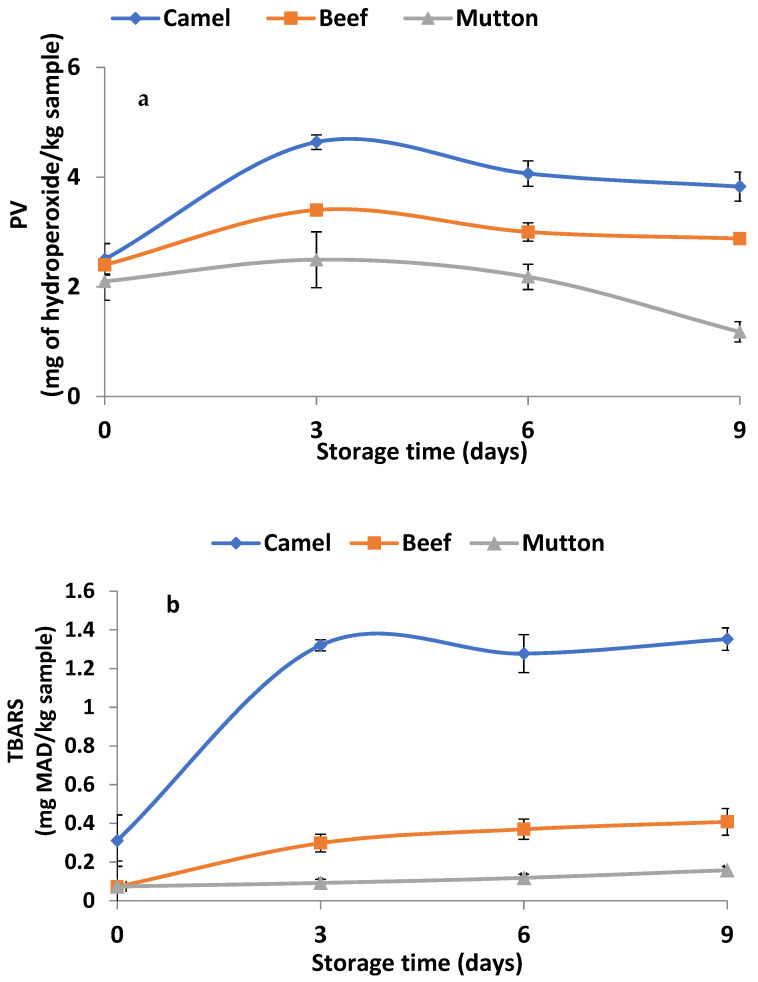
Changes in lipid oxidation products (peroxide value (**a**) and TBARS (**b**)) in camel meat, beef and mutton during 9 days of refrigerated storage. Data are represented as mean ± SEM (*n* = 6).

The peroxidase values in fresh camel meat and fresh beef were significantly (*p <* 0.05) higher than those in fresh mutton (Figure 3a). The storage of the meat samples for 3 days increased the PV of both the camel meat and beef samples, where the camel meat had the highest PV. However, the PV in the meat samples started to decrease as the storage period increased from day 3 to day 9, but the camel meat had the highest PV as compared with beef and mutton at the end of the 9-day storage period (*p* < 0.05). The fresh camel meat displayed a significantly higher TBARS value than fresh beef or fresh mutton did (Figure 3b) (*p* < 0.05). During storage, a sharp rise in the TBARS value in camel meat was observed in the first 3 days of storage (*p* < 0.05), thus indicating its high susceptibility to lipid oxidation. However, beef showed a gradual increase in the TBARS value from day 3 to day 9 of storage (*p* > 0.05). As storage progressed, there was variation in the rate of change in the TBARS in camel meat, while a slight increase in the TBARS value was noticed in beef (*p* > 0.05). Unlike other meat samples, there was no change in the TBARS value of mutton during storage (*p* > 0.05).

### 3.7. Changes in Total Pigment, Haemoglobin and Myoglobin

**Figure 4:**
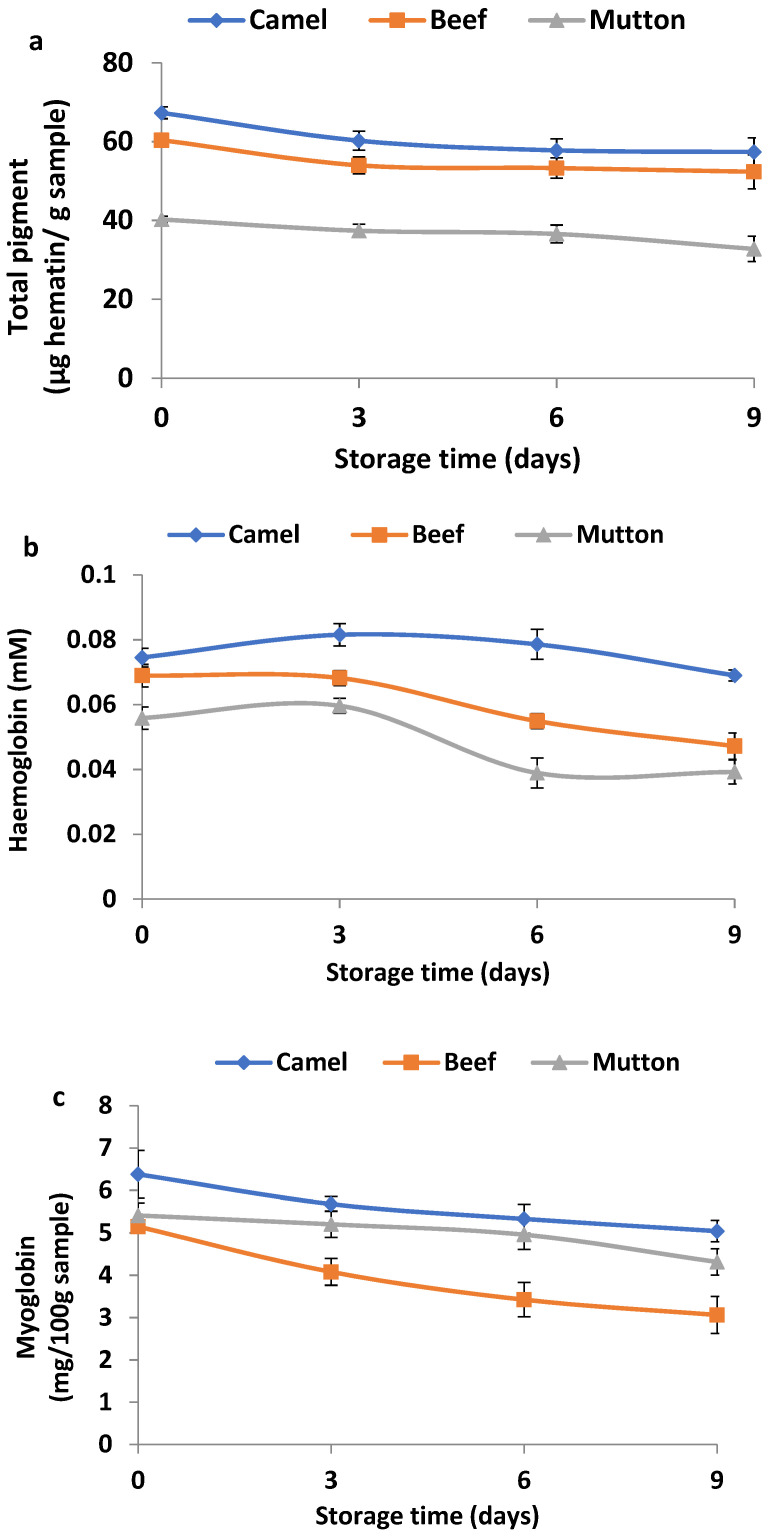
Changes in total pigment (**a**), haemoglobin content (**b**) and myoglobin content (**c**) in camel meat, beef and mutton during 9 days of refrigerated storage. Data are represented as mean ± SEM (*n* = 6).

**Figure 5:**
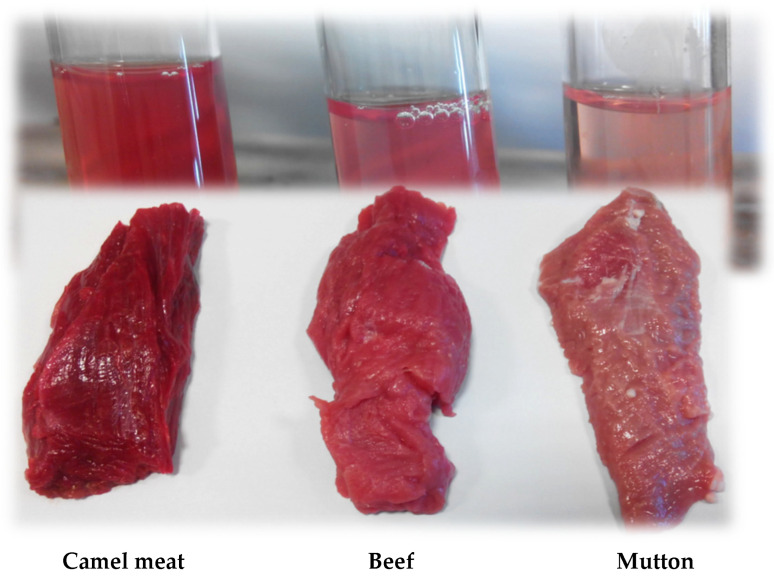
A picture showing meat chunks from camel, beef and mutton and their extracted haem pigments.

Fresh camel meat had a higher total haem content (67.32 μg haematin/g of sample) than beef (60.38 μg haematin/g of sample) or mutton (40.25 μg haematin/g of sample) (Figure 4a). The total haem content in camel meat and that in beef significantly (*p <* 0.05) decreased during the first 3 days of storage, and thereafter, the values were similar as storage progressed (*p* > 0.05). The fresh camel meat and beef samples had similar haemoglobin contents (*p* > 0.05), and the values were significantly higher (*p* < 0.05) than that of fresh mutton (Figure 4b). However, the fresh beef and fresh mutton had lower myoglobin contents than camel meat (Figure 4c) (*p* < 0.05). The haemoglobin contents in the meat samples started to decrease from day 3 of storage, with a sharp decrease observed in beef and mutton (*p* < 0.05). However, a gradual decrease in the myoglobin contents as the storage period increased until day 9 was noticed in all the meat samples. The total haem value plays a significant role in the colour of fresh meat (Figure 5).

### 3.8. Changes in Textural Properties

Table 2 compares the textural properties of the meat samples at day 0 and day 9 of refrigerated storage. On day 0 of storage, camel meat had significant values (*p <* 0.05) and the highest values for hardness, gumminess, chewiness and shear force, followed by beef, while mutton had the lowest values. All the measured textural parameters decreased from day 0 to day 9 of storage. A comparison of the meat samples on day 9 of storage showed that mutton had significant lower values (*p <* 0.05) for hardness and that camel meat had the lowest values for gumminess, respectively. Moreover, camel meat had the highest chewiness and shear force values compared with beef and mutton.

## 4. Discussion

The pH of meat is highly important because it has a major influence on other physicochemical and quality properties, such as water-holding capacity, tenderness and juiciness. The pH values of the meat samples in this study showed higher values (6.34–6.54) compared with other studies on beef (pH = 5.50) [23] and sheep (pH = 5.60) [24] meat at day 0. The variation in the pH values of camel meat during storage could be attributed to the high gluconeogenesis capacity of camels due to the presence of a camel hump, and the number of enzymes in its glycolytic pathway causes slower glycogen degradation and thus pH decline [8]. Similar to the pH of beef in this study, a nonsignificant change in pH for coarsely and finely marbled grade 1^+^ Hanwoo beef loins was observed during 14 days of storage at 2 °C [23]. Changes in pH are caused by postmortem metabolism [25]. Moreover, the difference in the rate of change in small letters pH of small letters meat samples could be attributed to the glycogen storage in the muscle at the time of slaughter.

The increase in drip loss during storage might be due to a greater loss of small letters water-holding property of the muscle protein due to degradation, and most of the proteins found in drip are water-soluble and sarcoplasmic proteins [26]. Similarly, the drip loss in packed camel meat significantly increased as storage duration increased [27]. The higher drip loss in fresh camel meat and mutton could be related to the lower intramuscular fat found in camel meat and mutton as compared with beef [28]. Intramuscular fat could act as a barrier to prevent fluid from passing, so the higher the fat, the lower the fluid loss [29]. The drip loss of meat may contribute to the lower consumer acceptability of meat, due to the fewer taste constituents remaining, and may contribute to the shrinkage of meat [30]. Our findings on WSP and SSP extractability are similar to those reported in porcine longissimus muscle during storage [31].

The high redness and yellowness in fresh camel meat may be due to the highest amount of haem pigment and myoglobin contents found in fresh camel meat as compared with the other two meat types (Figure 4). Similarly, Kadim et al. [10] found that meat from the camel longissimus muscle had a greater b* value than that of beef. The redness (a*) value of the beef after storage would meet the desired consumer thresholds of 14.5 for beef [32]. However, the a* values of mutton before and after storage were less than the desired threshold of 14.4 for fresh lamb [33]. The decrease in the a* value during storage may be due to the oxidation of oxymyoglobin to form metmyoglobin [34]. During the postmortem storage of meat, myoglobin in the ferrous state (+2) is oxidised to form ferric (+3) metmyoglobin, resulting in a brownish-red meat [35]. In addition, the observed colour change was in agreement with the decrease in the total haem pigment and myoglobin content during the storage of the meat samples.

As shown in the protein profiles of the meat samples, the higher protein degradation could have occurred because of the protein oxidation during storage, which could have led to the fragmentation and lysis of structural proteins [12]. Generally, a mild degradation of the beef protein was revealed compared with that of camel and mutton meat. The degradation of troponin T occurred during the postmortem storage of beef and camel meat for a period of 7 days [8]. Our findings agree with this study [8] in that we have also observed a marked degradation of bands corresponding to troponin T in all the three meat types during 9 days of storage. The degradation in troponin T was related to protein degradation and increased tenderness during storage [36]. Additionally, proteolysis by endogenous proteases during extended storage is responsible for protein degradation and the tenderisation of meat [4].

Lipid oxidation is an important factor influencing the quality of meat by affecting its flavour, colour, texture and nutritive value. Similar to this study, a decrease in the PV during storage was reported in the PV of chicken leg and breast meat [37,38] during frozen storage. The rise in the PV in camel meat and beef may be due to the faster rate of peroxide formation during the first 3 days of refrigerated storage, followed by their degradation into secondary oxidation products during the extended storage period of 9 days. Also, the high PV found in the camel meat could be due to the higher haemoglobin and myoglobin contents of camel meat compared with those of beef and mutton [4] given that these iron-containing pigments have been found to be effective catalysts for lipid oxidation [18].

As compared with day 0, the increase in TBARS in camel meat and beef during storage may be due to the degradation of hydroperoxides into secondary oxidation products, especially aldehydes, in the later stages of lipid oxidation [39]. Moreover, camel meat is known to contain higher numbers of the myoglobin and other haem compounds that function as pro-oxidants to promote lipid oxidation [4]. Furthermore, the denaturation of haem protein leads to the release of iron, which acts as a strong pro-oxidant and accelerates lipid oxidation [40]. Therefore, the high iron contents in camel meat after 9 days of storage (Supplementary Table S1) could be responsible for its high lipid oxidation products compared with beef and mutton. Similarly, a direct proportionality between haem pigment degradation and an increase in lipid oxidation during the storage of camel meat has been reported [4].

The variation in the total haem contents during storage could be due to the denaturation and oxidation of the pigment during storage, leading to the lower pigment content remaining or bound in the muscle [41]. Similar findings have been reported in raw minced sheep [42] and sardine and mackerel fish muscle [41] during refrigerated storage for 14 and 15 days, respectively. The oxidation of haem leads to the deep-yellow or brown discoloration of meat. The decrease in the total pigment corroborates well with the decrease in redness (a*) during storage from day 0 to day 9. The haemoglobin and myoglobin contents found in camel meat are comparable with those reported in a previous study [4] on camel meat, but the values were higher than those of beef and mutton found in the present study. The loss of myoglobin in the meat samples during storage could be due to its oxidation or degradation and loss of sarcoplasmic fluid (i.e., purge) [43]. Furthermore, haemoglobin and myoglobin are known to be potent catalysts of lipid oxidation in muscle foods [34]. Therefore, the high haemoglobin and myoglobin contents in camel meat may contribute to the rate of lipid oxidation in the camel meat sample during storage.

A decrease in the textural properties of all the meat samples as storage progressed may be attributed to the structural breakdown of myofibrils [44] and the degradation of meat proteins during the course of refrigerated storage, as shown in the SDS-PAGE profile. Furthermore, the proteolytic action promoted by muscle endopeptidases, resulting in tenderising the meats during 9 days of refrigerated storage, could not be ruled out. Similar to this study, camel meat was perceived to be tough and fibrous in [12]. Meat tenderness is greatly influenced by species, which could be due to differences in the coarseness of their musculature [45]. Furthermore, the shear force values in animal muscles have been reported to increase with the increasing age of the animal [10], and this was attributed to the changes in the connective tissues of the animals as the animals mature [46]. Therefore, the high shear force values in camel meat and beef in this study may be due to the old age of the animals as compared with the younger age of the animals from which the mutton samples were taken. Tenderness is an important quality attribute of meat, and therefore, suitable cooking methods should be selected for the cooking of the meats, particularly for stored camel meat, to improve the tenderness of the meat.

## 5. Conclusions

This study provided a characterisation of the protein and lipid fractions of three commercially important red meat (camel meat, beef and mutton), as well as changes occurring in their physicochemical and meat-quality attributes during 9 days of refrigerated storage. Camel meat was found to be highly susceptible to lipid oxidation during storage, and this may have been due to its high haem (haemoglobin and myoglobin) content and high percentage of polyunsaturated fatty acids. Protein extractability was found to be higher in mutton species, while protein solubility did not show any difference among the three animal species investigated. Increased drip loss was reported in the camel and mutton samples during the storage period. Camel meat was tougher, with higher hardness values, followed by the beef and mutton samples’ values, which decreased during 3 days and 9 days of storage, indicating the degradation of structural proteins. In conclusion, the study provided valuable information on the properties of different red meats during storage and variations in their protein and lipid fractions and other quality attributes during storage.

## Figures and Tables

**Table 1:** Changes in protein extractability and solubility, and colour properties of camel meat (CM), beef (BF) and mutton (MT) over 9 days of refrigerated storage.

Parameters	Sample	Storage Time (Days)			
		0	3	6	9
Protein extractability (Water-soluble protein) (mg of protein/g of sample)	CM	3.13 ± 0.01 ^bB^	3.51 ± 0.01 ^bA^	4.00 ± 0.00 ^bA^	4.04 ± 0.05 ^bA^
	BF	3.44 ± 0.01 ^aC^	3.48 ± 0.02 ^bC^	4.58 ± 0.01 ^aB^	4.86 ± 0.01 ^aA^
	MT	3.41 ± 0.02 ^aC^	4.35 ± 0.02 ^aB^	4.58 ± 0.01 ^aAB^	4.76 ± 0.01 ^aA^
Protein extractability (Salt-soluble protein) (mg of protein/g of sample)	CM	1.35 ± 0.02 ^bC^	1.67 ± 0.02 ^bB^	2.02 ± 0.01 ^aA^	2.06 ± 0.0 ^cA^
	BF	1.40 ± 0.02 ^bC^	1.87 ± 0.01 ^bB^	1.98 ± 0.01 ^aB^	2.59 ± 0.02 ^bA^
	MT	1.96 ± 0.01 ^aC^	2.18 ± 0.02 ^aB^	2.18 ± 0.01 ^aB^	3.01 ± 0.02 ^aA^
Sarcoplasmic protein solubility (mg of protein/g of sample)	CM	3.83 ± 0.02 ^bC^	3.97 ± 0.06 ^bC^	4.56 ± 0.01 ^cB^	5.06 ± 0.02 ^cA^
	BF	3.78 ± 0.01 ^bB^	3.95 ± 0.02 ^bB^	5.19 ± 0.01 ^bA^	5.36 ± 0.02 ^bA^
	MT	4.96 ± 0.02 ^aD^	5.21 ± 0.02 ^aC^	5.42 ± 0.01 ^aB^	5.81 ± 0.16 ^aA^
Total protein solubility (mg of protein/g of sample)	CM	9.86 ± 0.11 ^bB^	11.88 ± 0.05 ^aA^	12.03 ± 0.09 ^bA^	12.62 ± 0.02 ^bA^
	BF	10.17 ± 0.05 ^bB^	11.59 ± 0.07 ^aB^	13.12 ± 0.02 ^aA^	13.61 ± 0.03 ^aA^
	MT	11.12 ± 0.03 ^aC^	11.69 ± 0.07 ^aBC^	12.52 ± 0.01 ^abAB^	12.79 ± 0.02 ^abA^
L*	CM	41.69 ± 0.24 ^cA^	40.91 ± 0.38 ^cA^	35.61 ± 0.66 ^bB^	34.55 ± 0.73 ^bB^
	BF	45.58 ± 0.26 ^bA^	43.88 ± 0.08 ^bB^	42.16 ± 0.20 ^aC^	39.67 ± 0.13 ^aD^
	MT	46.84 ± 0.05 ^aA^	46.27 ± 0.03 ^aA^	43.38 ± 0.22 ^aB^	41.31 ± 0.12 ^aC^
a*	CM	21.67 ± 0.18 ^aA^	18.13 ± 0.39 ^aB^	12.43 ± 0.22 ^aC^	17.74 ± 0.20 ^aB^
	BF	18.74 ± 0.32 ^bA^	15.98 ± 0.13 ^bB^	14.85 ± 1.83 ^bD^	14.73 ± 0.15 ^bC^
	MT	13.84 ± 0.28 ^cA^	11.43 ± 0.09 ^cC^	10.18 ± 0.12 ^bD^	12.59 ± 0.12 ^cB^
b*	CM	17.10 ± 0.22 ^aA^	16.34 ± 0.13 ^aA^	11.05 ± 0.26 ^cB^	9.945 ± 0.14 ^cC^
	BF	16.97 ± 0.24 ^aA^	16.40 ± 0.07 ^aA^	14.74 ± 0.16 ^aB^	14.56 ± 0.13 ^aB^
	MT	15.82 ± 0.19 ^bA^	15.27 ± 0.03 ^bA^	13.08 ± 0.07 ^bB^	12.32 ± 0.15 ^bC^

Values are mean ± SEM (*n* = 9). Different small letters within the same column under the same tested parameter indicate significant differences (*p <* 0.05). Different capital letters within the same row under the same parameter indicate significant differences (*p <* 0.05).

**Table 2:** Changes in the textural properties of camel meat (CM), beef (BF) and mutton (MT) on day 0 and day 9 of refrigerated storage.

Storage Time	Samples	Hardness (N)	Cohesiveness	Springiness (mm)	Gumminess (g)	Chewiness (mJ)	Shear Force (N)
**Day 0**	**CM**	37.26 ± 0.08 ^a^	0.85 ± 0.08 ^a^	6.99 ± 0.08 ^a^	1906.3 ± 5.06 ^a^	98.12 ± 0.08 ^a^	58.05 ± 3.04 ^a^
	**BF**	30.49 ± 0.06 ^b^	0.89 ± 0.06 ^a^	6.97 ± 0.12 ^a^	1701.8 ± 5.92 ^b^	72.97 ± 0.05 ^b^	49.22 ± 1.66 ^b^
	**MT**	27.06 ± 0.07 ^d^	0.83 ± 0.04 ^ab^	6.91 ± 0.10 ^a^	1182.4 ± 3.72 ^c^	51.63 ± 0.07 ^d^	45.40 ± 1.17 ^c^
**Day 9**	**CM**	28.59 ± 0.05 ^c^	0.72 ± 0.04 ^c^	5.88 ± 0.09 ^b^	945.1 ± 3.23 ^e^	52.69 ± 0.06 ^d^	51.48 ± 1.96 ^b^
	**BF**	28.43 ± 0.04 ^c^	0.76 ± 0.03 ^bc^	5.84 ± 0.07 ^b^	992.7 ± 3.46 ^d^	47.48 ± 0.06 ^c^	41.58 ± 1.96 ^cd^
	**MT**	26.25 ± 0.03 ^e^	0.72 ± 0.02 ^c^	5.78 ± 0.03 ^b^	1178.3 ± 2.45 ^f^	46.54 ± 0.04 ^c^	38.73 ± 1.96 ^d^

Values are mean ± SEM (*n* = 6). Different small letters in the same column for each parameter on day 0 and day 9 of storage indicate significant differences (*p <* 0.05).

## Data Availability

Not applicable.

## Supplementary Materials

The following supporting information can be downloaded at https://www.mdpi.com/article/10.3390/ani13050904/s1; Table S1: Mineral composition of camel meat, beef and sheep meat at day 9 of storage.
