# Supplementary material for: A Comparative Study on Changes in Protein, Lipid and Meat-Quality Attributes of Camel Meat, Beef and Sheep Meat (Mutton) during Refrigerated Storage
Source: Animals (Basel). 2023 Mar 2;13(5):904. doi: 10.3390/ani13050904 (PMC10000245; doi:10.3390/ani13050904)
Supplement: Supplementary file 1 [file animals-13-00904-s001.zip › animals-2118697-supplementary.pdf]

Table S1. Mineral composition of camel meat, beef and lamb meat during 9 days of refrigerated storage

Sample/ Mineral content (ppm)	Ca	K	Mg	Na	S	P	Cu	Co	Fe	Mn	Zn
Camel Meat (CM)	484.2	2601.4	202.9	453.1	1785.4	1843.7	0.593	<0.003	30.4	0.043	56.7
Beef (BF)	78.4	2763.3	248.3	420.3	2019.6	1907.2	0.724	<0.003	15.1	0.101	30.5
Mutton (MT)	57.2	2601.2	235.1	617.7	2016.6	1890.1	1.067	<0.003	12.4	0.078	29.3
